# Le chordome sacrococcygien: à propos d’un cas

**DOI:** 10.11604/pamj.2021.40.164.30316

**Published:** 2021-11-17

**Authors:** Mohamed Moukhlissi, Ahmed Ben Sghier, Mohammed Bensaid, Loubna Mezouar

**Affiliations:** 1 Département de Radiothérapie, Centre Hospitalier Universitaire Mohammed VI, Faculté de Médecine et de Pharmacie, Université Mohammed 1er, Oujda, Maroc,; 2 Laboratoire BENSAID d´Anatomie-Pathologique, Oujda, Maroc

**Keywords:** Chordome, sacro-coccyx, douleur, radiothérapie, à propos d’un cas, Chordome, sacrococcyx, pain, radiotherapy, a case report

## Abstract

Le chordome sacrococcygien est une entité pathologique rare, rapporté dans seulement moins de 3% des cas. Nous allons rapporter l´observation d´une patiente prise en charge au Centre Régional d'Oncologie d'Oujda au Maroc ayant présenté un chordome sacrococcygien localement avancé. Le traitement a consisté en une radiothérapie exclusive sur la tumeur, la chirurgie d´exérèse n´était pas possible. Actuellement la patiente est à 2 ans de recul, la tumeur est stable sur le plan clinique et radiologique avec un bon état général.

## Abstract (en)

Sacrococcygeal chordoma is a rare disease. It has been described in less than 3% of cases. We here report the case of a female patient with locally advanced sacrococcygeal chordoma treated at the Oujda Regional Oncology Center in Morocco. The treatment was based on exclusive radiotherapy; surgical resection couldn’t be performed. At 2-year follow-up the tumor is clinically and radiologically stable and general condition of the patient is good.

## Introduction

Les chordomes sont des tumeurs osseuses primitives rares développées aux dépens des reliquats de la notochorde embryonnaire [1]. Ce sont des tumeurs à croissance lente, elles envahissent les structures avoisinantes en détruisant les os, les nerfs, et les autres organes de voisinage, et donnent rarement de métastases à distance cependant elles présentent un haut risque de récidive local et locorégional après le traitement [2]. Elles siègent souvent aux deux extrémités de névraxe: la base du crâne (35%-40%), le sacrum dorsal (40% à 50%) [3, 4]. Ce sont des tumeurs souvent pauci-symptomatique, le maitre symptôme d'un chordome sacré est la douleur. Le traitement optimal des chordomes est basé sur une résection chirurgicale large. La radiothérapie permet de retarder les récidives locales et de traiter la douleur, mais elle n´a aucun intérêt dans le traitement curatif sans association à la chirurgie [5] sauf dans un but palliatif antalgique ou décompressif quand la chirurgie n´est pas possible. Nous rapportons un cas clinique d´un chordome sacré traité au centre régional d´oncologie à Oudja dans le service de radiothérapie.

## Patient et observation

**Figure 1:**
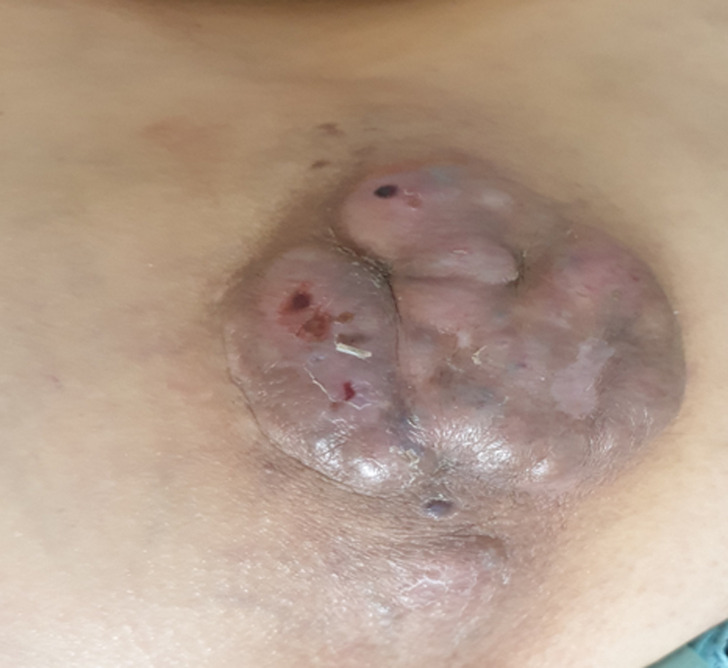
photo de la masse sacrée

**Information de la patiente:** une patient de 58 ans, sans antécédent pathologique notable, elle est admise en service de radiothérapie pour la prise en charge d´une masse sacro-coccygienne. La symptomatologie évoluait depuis un an sous la forme d´une petite tuméfaction cutanée sacrée, douloureuse, qui a augmenté progressivement de volume et s´est ulcérée par endroit (Figure 1). La patiente n´a aucun signe neurologique ou digestif et elle est en assez bon état général.

**Résultats cliniques:** l´examen clinique révèle une tuméfaction sacrée mesurant 8 cm sur 5 cm, de consistance molle, douloureuse et fixe par rapport au plan profond. Le toucher rectal objective l´existence d´une masse bombante dans le bas rectum respectant la muqueuse.

**Figure 2:**
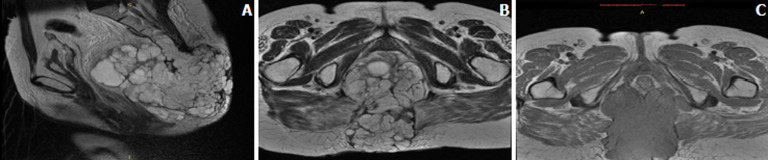
aspect IRM montrant une masse tissulaire polylobée, bien limitée, en regard du coccyx, refoulant en avant l'ampoule et le canal rectal. Cette masse est de consistance non graisseuse, se rehaussant après injection de produit de contraste, (A) T2 coupe sagittale, (B) T2 coupe axiale, (C) T1 coupe axiale

**Figure 3:**
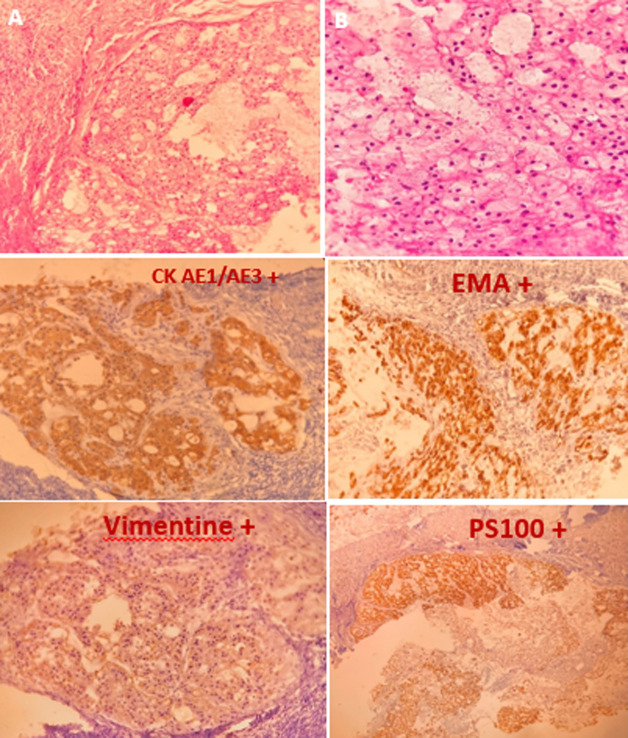
aspect anatomopathologique et immunohistochimique de la tumeur (A) HE X 20: prolifération tumorale en lobules composés de cellules claires de grande taille, (B) HE X 40: cellules au cytoplasme vacuolaire claire et aux noyaux arrondis central modérément atypique (cellule physalifores) IHC: CK AE1/AE3+, EMA+, Vimentine+, PS100+

**Démarche diagnostique:** la tomodensitométrie (TDM) et l´imagerie par résonnance magnétique (IRM) confirment qu´il s´agit d´une masse tissulaire de 14 cm de diamètre polylobée, bien limitée, en regard du coccyx, refoulant en avant l´ampoule et le canal rectal, sans signe d´envahissement de ceux-ci. Cette masse est de consistance non graisseuse, se rehaussant après injection intraveineuse de produit de contraste (Figure 2). La biopsie de la masse avec l´étude anatomopathologique et immunohistochimique ont permises de poser le diagnostic du chordome sacrococcygien (Figure 3). La tumeur était chirurgicalement non résécable.

**Intervention thérapeutique et suivi:** une radiothérapie a été réalisée sur la tumeur à une dose totale de 66 Gy en 33 fractions par radiothérapie conformationnelle. Après 2 ans d´évolution, la patiente est encore vivante avec moins de douleurs et la tumeur est d´aspect clinique et radiologique stable.

## Discussion

Le chordome est globalement une tumeur très rare. Son incidence varie entre 0,5 à 8 cas par million d´habitants et par an [6]. Il représente de 1 à 4% des tumeurs osseuses malignes primitives [2]. Le chordome peut se voir à tous les âges, avec un âge médian de diagnostic de 58,5 ans et l´incidence augmente progressivement avec l´âge [2]. L´homme est nettement plus atteint que la femme (sex-ratio H /F = 3/1) [7]. Pour notre cas, il s´agit d´une femme de 58 ans. Le chordome est une tumeur d´origine embryonnaire. Il résulte de la prolifération des ilots cellulaires persistants dans les vertèbres ou la base du crâne. Ces ilots cellulaires sont des vestiges embryonnaires.

L´examen anatomopathologie est un élément fondamental et indispensable pour le diagnostic des chordomes comme d´ailleurs pour toutes les tumeurs osseuses. Selon l´OMS: « les chordomes sont des tumeurs malignes présentant une différenciation notochordale ». Macroscopiquement, c´est une tumeur généralement lobulée, de consistance molle gélatineuse, de couleur grisâtre ou blanc-bleuté [2]. Microscopiquement, les cellules tumorales sont de grande taille, à cytoplasme bien délimité, éosinophile homogène ou clarifiées, contenant une ou plusieurs vacuoles optiquement vides refoulant le noyau, déformant parfois le cytoplasme réalisant l´aspect de cellule physaliphore ou de cellule araignée. Ces cellules sont groupées en amas cohésifs de taille variable, ou forment des travées monocellulaires, ou des cellules isolées, sur une matrice myxoïde d´abondance très variable d´un lobule tumoral à l´autre au sein de la même tumeur. Les noyaux ont une taille et un contour variable, un peu irrégulier et hyperchromatiques. Des mitoses, et des remaniements inflammatoires hémorragiques ou nécrotiques parfois extensifs, et de l´apoptose peuvent être observées [2]. Dans les chordomes, Les pancytokératines et la cytokératine 19, l´EMA (épithélial-membrane antigène) et la vimentine sont quasi constamment exprimés, mais parfois seulement de façon focale. Le brachyurie est un marqueur plus récent [8], très spécifique du chordome avec une sensibilité de l´ordre de 90,2% [2].

Pour notre cas, l´étude immunohistochimique: AE1/AE3: un marquage positif diffus des cellules tumorales; EMA: marquage positif focal des cellules tumorales; PS100 et vimentine: marquage positif focal des cellules tumorales. Les signes cliniques sont en fonction de la taille et du siège de la tumeur. Ils sont en général tardifs du fait de l´évolution lente de ces tumeurs et non spécifiques [7, 8]. Les signes cliniques sont souvent dominés [9] par la douleur, le signe révélateur le plus fréquent. Cette douleur est secondaire à la compression et/ou l´envahissement des organes de voisinage. Les troubles digestifs (syndrome rectal, constipation, syndrome occlusif…). Les troubles urinaires: pollakiurie, dysurie, incontinence urinaire… Les troubles neurologiques: qui sont secondaires à la compression ou à l'envahissement des différents plexus nerveux et racines nerveuses de la région. Dans 97% des cas, l'inspection du périnée permet de révéler la présence d'une tuméfaction fessière ou périnéale postérieure ou d´un comblement de la région présacrée évoquant une tumeur rétrorectale [10]. Le toucher rectal objective souvent une masse présacrée de consistance ferme, indolore, parfois lobulée fixée, rugueuse [8]. Il permet d´en apprécier la taille, le siège en hauteur par rapport à l´appareil sphinctérien et la limite supérieure, il permet aussi de contrôler la sensibilité périnéale pour éliminer une atteinte nerveuse [9]. Pour notre cas, le toucher rectal a révélé une sténose rectale par compression d´une masse postérieure.

Les techniques d´imagerie modernes occupent une place importante dans le bilan paraclinique du chordome sacré, permettant d´évoquer le diagnostic, la caractériser et surtout d´en étudier les rapports. Les clichés de l´abdomen sans préparation (ASP), ainsi que ceux centrés sur le bassin, de face et de profil: bien qu´il n´existe pas de signes radiologiques pathognomoniques, on considère en général que les chordomes comportent quatre grandes caractéristiques: expansion - raréfaction osseuse - trabéculations intra tumorales – calcifications [10]. La TDM revêt un très grand intérêt tant dans le bilan pré-thérapeutique que pour la surveillance [8], elle permet de préciser la nature de la masse, apprécier son caractère, sa topographie et ses limites, et éventuellement ses extensions locorégionales et à distance [10]. On décrit des destructions osseuses sacrococcygiennes associées à une masse tumorale qui souvent volumineuse à bords nets, dont la densité se rapproche de celle des tissus mous [11, 12]. L'IRM pelvienne à côté de la TDM apporte une grande précision topographique notamment pour les tissus mous, l´espace épidural et intra dural (atteinte des racines nerveuses) et permet d'apprécier la composante liquide ou solide de la lésion, sa contiguïté avec le rectum, ses limites, et par conséquent évoquer le diagnostic tout en déterminant le meilleur accès chirurgical [2]. L´intérêt de la tomographie par émission de positons (PET-scan) 18FDG réside dans le diagnostic différentiel entre chordome (captation nulle) et métastase (captation intense et parfois lésions multiples) [2].

Plusieurs lésions peuvent imiter un chordome sacré comme les chondrosarcomes, les tumeurs à cellules géantes, les kystes anévrysmaux, le sarcome d´Ewing, l’épendymome myxopapillaire, et les métastases osseuses [2, 8, 10], dont les données radiologiques, histologiques et immunohistochimique permettent de les écarter. La seule chance de guérison repose sur une résection chirurgicale « en bloc », avec des limites d´exérèse en zone saine [8]. Le taux de récidive tumorale reste malgré tout relativement élevé [2]. Ce type de résection est en général plus facilement réalisable lorsque la tumeur siège au-dessous des 3 premières vertèbres sacrées. On sait par ailleurs que les sacrectomies étendues au-dessus de S3, incluant une partie des articulations sacro-iliaques, sont techniquement difficiles et entraînent fréquemment des complications, notamment des troubles sphinctériens et des troubles de la marche [12]. Ces exérèses sont parfois difficiles et hémorragiques, en cas de grosses tumeurs, de ce fait elles doivent respecter la stabilité du pelvis et d´éviter les complications neurologiques. Pour réaliser une résection large et en bloc, il est le plus souvent proposé un abord postérieur pour les chordomes ne dépassant pas S3. En revanche, un abord combiné antérieur et postérieur est utilisé pour ceux dépassant S3 [13].

La radiothérapie trouve sa place soit en adjuvant après la chirurgie, soit exclusivement en cas de récidive locale, voire, lorsque la chirurgie est impossible [13]. Devant les taux de rechute élevés obtenus après une chirurgie exclusive, il a été initialement proposé une irradiation adjuvante par photons, cependant La survie sans progression était toujours inférieure à 40%. La proximité des organes à risque par rapport à la tumeur et les propriétés balistiques des photons empêchaient souvent de délivrer des doses supérieures à 60 Gy au niveau de la tumeur, ce qui impactait directement le contrôle local [2]. Les chordomes ont une radiosensibilité variable, mais dans la plupart des cas il s´agit des tumeurs radio-résistantes [14]. Ceci fait discuter l´indication d´une radiothérapie adjuvante qui trouve sa place après une chirurgie R1 et R2 à la dose de 50 à 60 Gy en 5 à 6 semaines. La radiothérapie est parfois indiquée de manière exclusive et palliative, en cas de très volumineuses tumeurs non opérables. Une première série d´irradiation externe à visée décompressive et antalgique est délivrée jusqu´à une dose de 50 GY en 5 semaines. En cas de bonne réponse tumorale objective à l´examen tomodensitométrique de contrôle, on peut effectuer un complément d´irradiation externe de 20 GY en 2 semaines.

Une irradiation externe exclusive n´entraine qu´exceptionnellement une destruction tumorale complète, en revanche, on obtient souvent un bon confort et surtout un bon effet antalgique. Etant donné la possibilité de survenue des métastases, des combinaisons chimiothérapiques ont été essayées malheureusement sans succès [15]. La protonthérapie permet d´augmenter la dose dans la tumeur et d´épargner au maximum les organes critiques avoisinants grâce aux caractéristiques balistiques des protons [2]. Cette particularité physique est fondamentale pour expliquer le gradient de dose qui peut être obtenu à proximité d´un organe critique. La dose varie de 10 à 15% par millimètre de tissu traversé [16]. Il a alors été montré que le contrôle local était amélioré et le risque de toxicité acceptable dans de très nombreuses séries. C´est pourquoi, depuis plusieurs années, l´irradiation par protons est devenue la technique d´irradiation de référence dans la prise en charge des chordomes de la base du crâne après chirurgie [16]. L´utilisation des ions carbone représente une modalité intéressante. En effet, ils possèdent les avantages physiques des protons (pic de Bragg) et une efficacité biologique relativement supérieure, intéressante pour les tumeurs radio résistantes. Des résultats prometteurs ont été obtenus au Japon et en Allemagne en termes d´efficacité et aussi en termes de toxicité [14, 17].

L´expérience de la chimiothérapie s´est révélée le plus souvent décevante, mais il semble toutefois utile de l´employer dans les localisations secondaires de la maladie [2]. En pratique, la chimiorésistance de ces tumeurs est reconnue même si des résultats positifs et isolés ont pu être rapportés avec les anthracyclines, les agents alkylants, le cisplatine et la thalidomide [2, 18]. Egalement l´imatinib seul ou associé au sirolimus a montré une certaine efficacité en terme de contrôle locale dans les chordomes exprimant le PDGF [19, 20]. Le pronostic des Chordomes sacrés dépend surtout de la qualité de l´exérèse chirurgicale. En dehors des complications en relation avec l´acte chirurgical lui-même, des complications secondaires peuvent survenir telles les récidives locales qui sont un évènement péjoratif réduisant significativement la survie globale des patients [2]. L´intérêt de la radiothérapie reste débattu car la majorité des séries de cas n´a pas révélé d´effet majeur sur la survie [15]. À ce jour, la radiothérapie est adaptée pour contrôler localement la tumeur mais la capacité réelle de la Radiothérapie adjuvante à améliorer la survie sans récidive et globale demeure inconnue. La moyenne de survie, dans la série d´Erikson était de 6,6 années pour le groupe Radio chirurgie, contre 5,7 années pour la chirurgie seule et 5,4 années pour la radiothérapie seule. Dans notre cas, après 2 ans de la fin de radiothérapie, la patiente est encore vivante avec moins de douleurs et une stabilité clinique et radiologique de la tumeur.

## Conclusion

Le chordome sacré de l'adulte constitue une entité rare. Ils sont développés à partir des résidus embryonnaires, des restes de notochorde. Le diagnostic est très tardif du fait de la latence clinique dont la douleur représente le maitre symptôme. Le diagnostic positif est basé sur la clinique et l´imagerie avec la confirmation par l´examen anatomopathologique. Le traitement de base est chirurgical, et doit consister en une exérèse totale dans la mesure du possible afin d´éviter à la fois les complications et les récidives. La radiothérapie intervient à titre de complément de la chirurgie, d´emblée ou en cas de récidive locale, voire une radiothérapie exclusive, lorsque la chirurgie est impossible. Le chordome sacré se caractérise par son potentiel malin à haut risque de récidive local et locorégional, en revanche les métastases sont tardives.
